# The Circadian NAD^+^ Metabolism: Impact on Chromatin Remodeling and Aging

**DOI:** 10.1155/2016/3208429

**Published:** 2016-12-05

**Authors:** Yasukazu Nakahata, Yasumasa Bessho

**Affiliations:** Laboratory of Gene Regulation Research, Graduate School of Biological Sciences, Nara Institute of Science and Technology (NAIST), 8916-5 Takayama, Ikoma, Nara 630-0192, Japan

## Abstract

Gene expression is known to be a stochastic phenomenon. The stochastic gene expression rate is thought to be altered by topological change of chromosome and/or by chromatin modifications such as acetylation and methylation. Changes in mechanical properties of chromosome/chromatin by soluble factors, mechanical stresses from the environment, or metabolites determine cell fate, regulate cellular functions, or maintain cellular homeostasis. Circadian clock, which drives the expression of thousands of genes with 24-hour rhythmicity, has been known to be indispensable for maintaining cellular functions/homeostasis. During the last decade, it has been demonstrated that chromatin also undergoes modifications with 24-hour rhythmicity and facilitates the fine-tuning of circadian gene expression patterns. In this review, we cover data which suggests that chromatin structure changes in a circadian manner and that NAD^+^ is the key metabolite for circadian chromatin remodeling. Furthermore, we discuss the relationship among circadian clock, NAD^+^ metabolism, and aging/age-related diseases. In addition, the interventions of NAD^+^ metabolism for the prevention and treatment of aging and age-related diseases are also discussed.

## 1. Introduction

In humans, each cell has a nucleus, the diameter of which is ~10 *μ*m, packing approximately 2 m long DNA if stretched end to end. DNA in the nucleus is arranged at several units, from chromatin to chromosome [1, 2]. Chromosomes in the nucleus are known to be present nonrandomly, located at the spatially confined regions; for example, euchromatin prefers center and heterochromatin periphery of nucleus [2, 3]. Despite their relatively low abundance in the cell, transcription factors have to search for their target gene, usually existing in only 2 copies per cell, to activate or repress in such a spatially messy condition [4]. Such conditions make gene expression stochastic [4]. Mechanical changes of nuclear events referred to as topological changes of chromosome and further epigenetic or posttranslational changes of chromatin have been suggested to increase or decrease stochastic gene expression rate. Changing topology of chromosomes, by receiving soluble factors and also by sensing the extracellular microenvironment, can lead stem cells to differentiate into multiple cell types [1, 2, 5–8]. Alteration of gene expression program by topological changes contributes not only to development/differentiation, but also to pathophysiological conditions such as cancer, diabetes, and aging [9].

Epigenetic events are mediated by chemical modifications of DNA and/or histones. In particular, histone modifications, such as acetylation, phosphorylation, methylation, and ubiquitination, bring about transient, nonheritable modifications in the genome, which increase or decrease the stochastic gene expression rates [10, 11]. These modifications alter stochastic gene expression rates by changing the property of chromatin surface. For example, the histone modification that involves the addition of an acetyl group neutralizes the negatively charged lysine, releasing DNA from histone, thereby facilitating recruitment of transcription activators to DNA. Many enzymes have been identified as chromatin remodeling factors such as CBP/p300 and PCAF (histone acetyltransferases, HAT), HDACs and SIRTs (histone deacetylases), SUV39H1, and MLLs (histone methyltransferases). Epigenetic modifications of chromatin are known to be closely associated with fate specifications of stem cells or precursor cells during embryogenesis [12]. On the other hand, differentiated cells in the adult body are also known to carry out their functions as a result of altered chromatin structure. One example of this is the rhythmic 24-hour cell-autonomous chromatin remodeling that occurs within a cell.

In this review, we discuss data suggesting that chromatin structure changes in a circadian manner and that NAD^+^ is the key metabolite for circadian chromatin remodeling. Furthermore, we discuss the relationship between circadian clock, NAD^+^ metabolism, and aging/age-related diseases. In addition, the interventions of NAD^+^ metabolism for the prevention and treatment of aging and age-related diseases are also discussed.

## 2. Circadian Clock

The earth's rotation period is 23 hours, 59 minutes, 4.1 seconds, or approximately 24 hours. To adjust themselves to the earth's rotation, almost all living things, including cyanobacteria, insects, fish, and mammals, have developed their endogenous clock systems, which are referred to as the “circadian clock.” In mammals, circadian clock exists in all tissues of the body, including each cell [13], although there is a hierarchy among clocks. A master clock is located at the suprachiasmatic nucleus (SCN) of the anterior hypothalamus of the brain [13]. The SCN is composed of a pair of tiny nuclei (10,000 cells in each sphere), and each cell has its own circadian clock machinery and is thereby able to drive circadian rhythm cell-autonomously. The SCN receives daily light/dark information via the retinohypothalamic tract in order to entrain to the environmental conditions and transmits this information to peripheral clocks present in all other tissues in the body through hormone secretions and neuronal innervations. Although signals from the SCN are the dominant cues to entrain circadian clock in peripheral tissues, other cues such as nutrition, exercise, or pathophysiological conditions can entrain or remodel peripheral clocks [14–18]. In order to perform tissue-specific and/or interorgan functions with 24-hour rhythmicity in the body, more than 10% of all transcripts show circadian changes, and many nonoscillatory transcripts also demonstrate oscillations at protein levels due to posttranscriptional regulations or at enzymatic activity levels such as casein kinase and SIRT1 [17–23]. An interactive network of master and peripheral circadian clocks regulate many metabolic and homeostatic balances to preserve multiorgan networks, thereby keeping the organism healthy.

## 3. Molecular Clock

**Figure 1:**
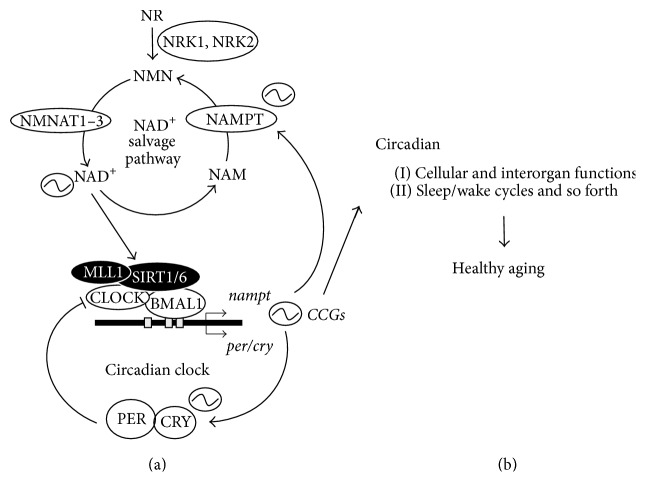
Importance of circadian clock in maintaining health and healthy aging. (a) CLOCK and BMAL1 activate an array of genes by binding to E-box elements on their promoter.* Per* and* Cry* transcripts are two of the target genes and PER/CRY repress CLOCK/BMAL1 activity to drive 24-hour rhythmicity (circadian rhythm).* CCGs* including* nampt* transcripts show circadian oscillations to maintain cellular, interorgan, and systemic functions, thereby contributing to healthy aging (b). CCGs: clock-controlled genes; NAD^+^: nicotinamide adenine dinucleotide; NAM: nicotinamide; NMN: nicotinamide mononucleotide; NR: nicotinamide riboside; NAMPT: nicotinamide phosphoribosyltransferase; NMNAT1~3: NMN adenylyltransferase 1, NMN adenylyltransferase 2, and NMN adenylyltransferase 3; NRK1 and NRK2: nicotinamide ribose kinase 1 and nicotinamide ribose kinase 2.

Molecular mechanisms of circadian clock in many model animals, such as* Drosophila melanogaster*,* Neurospora crassa*, and mammals, have been dissected, revealing that a wide array of underlying molecules are vital for circadian clocks to maintain circadian rhythmicity. Today, a complex network of interlocked transcriptional-translational feedback loops constitute a universal system of the molecular clocks in all model animals examined to date [24]. In* Drosophila melanogaster*, for instance, transcriptional factors, CLOCK and CYCLE, activate* period* and* timeless* gene expressions, and PERIOD and TIMELESS proteins repress their own transcriptions, thereby making a transcriptional negative feedback loop. This system is very similar to mammalian and other circadian clock systems, although molecules are not evolutionarily conserved [24]. Molecular mechanisms that make up the core circadian clock in mammals consist of transcriptional factors, CLOCK, NPAS2, BMAL1, PERIOD1-2 (PER1-2), CRYPTOCHROME1-2 (CRY1-2), ROR, and REV-ERB [25]. CLOCK, NPAS2, and BMAL1 are bHLH-type transcriptional activators. BMAL1 makes a heterodimer with CLOCK or NPAS2 to activate gene expressions by binding to E-box element on the promoter of target genes.* Pers* and* Crys* are two of the CLOCK:BMAL1/NPAS2 target genes and PERs and CRYs can also heterodimerize and give feedback to repress their own transcription by binding to CLOCK:BMAL1/NPAS2 (Figure 1). However, since this repression is transient due to the decrease of* Pers and Crys* mRNA, repression of CLOCK:BMAL1/NPAS2 activity is released and the next cycle of this feedback loop can begin. The nuclear receptors, ROR and REV-ERB, recognize and compete for binding to the same element, RORE (ROR/REV-ERB-response element), to serve as an activator and a repressor, respectively, at target sites to drive peripheral transcriptional-translational loop that impinges on the core clock feedback loop.

## 4. Circadian Metabolites

As indicated, a wide array of cellular transcripts and proteins demonstrate a circadian pattern in many tissues, not only under the normal condition, but also under environmental or pathophysiological conditions [15, 17, 18]. For instance, high-fat diet (HFD) induces a large number of* de novo* oscillating transcripts in the mouse liver [18]. Furthermore, gene ontology analyses have revealed that a series of genes associated with metabolic processes show periodic expression with a 24-hour rhythm under both normal and pathophysiological conditions [17, 18]. Metabolomics analyses have confirmed that many metabolites, including lipids, polyamines, amino acid and glucose/glycolytic intermediates, and nicotinamide adenine dinucleotide (NAD^+^; discussed in detail later), S-adenosylmethionine (SAM), and acetyl-CoA, demonstrate a 24-hour oscillation in many tissues and cultured cells [17, 18, 26–31]. Among circadian metabolites, acetyl-CoA and SAM are acetyl and methyl donor, respectively, for histone and nonhistone proteins. Interestingly, NAD^+^, another circadian metabolite, is a cosubstrate for Sirtuin family protein deacetylase, suggesting that proteins, especially histone proteins, can undergo circadian modifications in the abundance of acetyl and methyl groups.

## 5. Circadian Chromatin Remodeling

**Figure 2:**
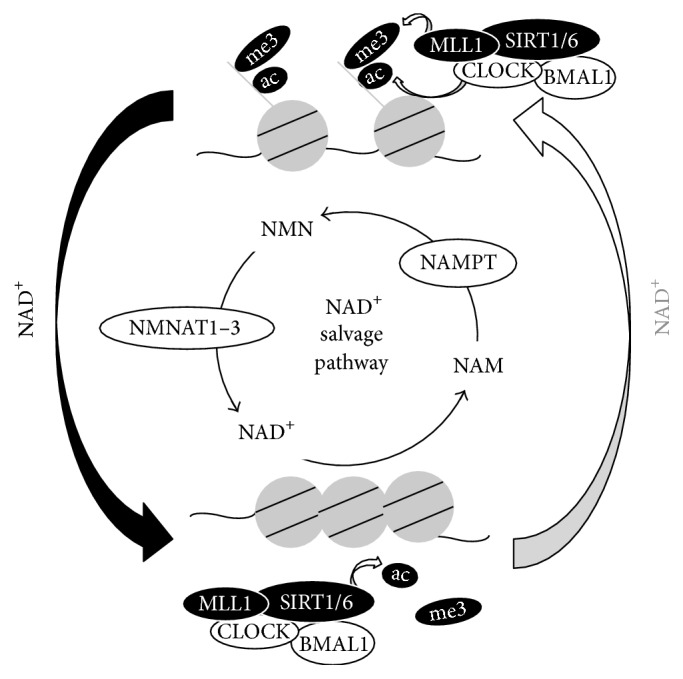
Circadian NAD^+^ metabolism modifies chromatin properties with a 24-hour rhythm. Circadian NAD^+^ metabolism controls histone modifications through acetylation and methylation processes, therefore regulating precise circadian gene expression. NAD^+^ with black arrow represents high NAD^+^ amount and NAD^+^ with gray arrow signifies low NAD^+^ amount.

Chromatin remodeling contributes largely to the regulation of gene expression in many systems [10, 32, 33], and the regulation of gene expression by circadian clock is no exception. Circadian acetylation of histone H3 lysine 9 and 14 (H3K9/14ac) was first reported to demonstrate circadian change on promoters of E-box-regulated circadian clock genes,* per1*,* per2*, and* cry1* [34]. Although p300, one of HAT, was proposed in that report to be a putative HAT for circadian H3K9/14ac, a later report demonstrated that CLOCK itself also possesses HAT activity and preferentially acetylates H3K9/14 [35]. Subsequently, it was reported that SIRT1, an NAD^+^-dependent Sirtuin family deacetylase, counteracts the HAT CLOCK [36].* Sirt1* ^−/−^ cells completely ablated circadian H3K9/14ac rhythm and altered circadian gene expressions, indicating that circadian changes of chromatin property are necessary for fine-tuning circadian gene regulations (Figures 1 and 2) [36]. SIRT1 is activated by many stimuli, including low nutrition, low ATP, and exercise, and consequently it has been described as an energy and nutrition sensor [37]. Thus, SIRT1 transduces signals originated by cellular metabolites to the circadian clock.

SIRT6, another Sirtuin family deacetylase, has also been shown to control gene expressions in a circadian way. Interestingly, although SIRT6 can form a complex with CLOCK:BMAL1 similar to SIRT1, its target genes are different [38]. Whereas SIRT1 shuttles between the nucleus and the cytoplasm and preferentially regulates genes related to peptides and cofactors metabolism, SIRT6 localizes constitutively in the nucleus, especially at the euchromatin regions, and controls genes implicated in lipids and carbohydrates metabolism. This suggests that genomic partitioning by two independent Sirtuins contributes to differential control of circadian metabolism.

While H3K9/14ac has been functionally associated with H3K4 trimethylation (H3K4me3), which is also associated with transcriptionally active genes [11], it was demonstrated in circadian clock machinery that H3K4me3 cycles with 24-hour period and follows the same phase as H3K9/14ac rhythm (Figure 2) [39]. MLL1, an H3K4-specific methyltransferase, was shown to be responsible for the rhythmic H3K4me3 and circadian gene expressions, indicating that circadian changes of chromatin property by H3K4me3 are also necessary for fine-tuning circadian gene regulations. Furthermore, it has been indicated that MLL1 is an acetylated protein and its deacetylation, following the inactivation is controlled by SIRT1 [40], suggesting that regulation of SIRT1/6 activity is the key for circadian changes of chromatin property by H3K9/14ac and H3K4me3.

## 6. Circadian NAD^+^ Metabolism

Since SIRT1 deacetylation activity exhibits a clear circadian oscillation, intracellular NAD^+^, which is a cosubstrate for its activity, was assessed by LC-MS^*n*^ methods. These experiments revealed that cellular NAD^+^ amount demonstrates circadian rhythm [30]. Significantly, this oscillation has been shown to be a key regulator of rhythmic H3K9/14ac and H3K4me3 chromatin marks [30, 40]. NAD^+^ is consumed by NAD^+^-consuming enzymes, Sirtuins, PARPs, and CD38. These enzymes deacetylate proteins, build poly-ADP-ribosylation, and generate cyclic ADP-ribose, respectively [41]. While NAD^+^/NADH redox reaction has no effect on the total amount of NAD^+^ and NADH, enzymatic reactions by NAD^+^-consuming enzymes do decrease NAD^+^, producing nicotinamide (NAM) as a common byproduct. NAM is recycled to produce NAD^+^ by the intracellular NAD^+^ salvage pathway (Figures 1 and 2), which is known to be dominant for intracellular NAD^+^ biosynthesis in many cells and tissues. NAM is converted to nicotinamide mononucleotide (NMN) by nicotinamide phosphoribosyltransferase (NAMPT), and then NMN is converted to NAD^+^ by nicotinamide mononucleotide adenylyltransferases 1–3 (NMNAT1–3) [42]. Among these enzymes, NAMPT is believed to be the rate-limiting enzyme for the NAD^+^ salvage pathway. Intriguingly, circadian gene expression analysis and* Nampt* promoter analysis have confirmed that* nampt* gene expression is under the regulation of circadian clock. Thus, intracellular NAD^+^ amount is regulated by circadian clock, oscillating with 24-hour rhythmicity [30] (Figures 1 and 2). Of note, intracellular NAD^+^ oscillation is in phase with SIRT1 deacetylase activity and in opposite phase of MLL1 methyltransferase activity, thereby showing that the phase of oscillatory NAD^+^ is opposite to that of acetylation and methylation of histone H3 (Figure 2). Interestingly, intracellular NAD^+^ in the liver of circadian clock deficient (*bmal1* ^−/−^ or* clock* ^Δ19/Δ19^) mice and in MEF cells derived from* bmal1* ^−/−^,* clock* ^Δ19/Δ19^, or* cry1* ^−/−^ */2* ^−/−^ mice were significantly reduced [30, 31], indicating that circadian clock controls basal and circadian NAD^+^ amount. Pharmacological experiments using FK866, an NAMPT inhibitor, reduced cellular NAD^+^ by at least 80% and demonstrated an altered pattern of circadian gene expression [30], while CD38 knockout mice, which have high cellular NAD^+^, also showed altered gene expression and locomotor activity patterns [43]. In addition, HFD, which remodeled circadian clock and completely ablated oscillations in cellular NAD^+^ in mouse liver, drove an altered circadian gene expression pattern [18]. These reports indicate that proper circadian NAD^+^ regulation is indispensable for the maintenance of circadian clock, suggesting that it is probably essential for preservation of health (discussed below).

## 7. NAD^+^ Metabolism and Aging

Although cellular NAD^+^ amounts fluctuate within a short period (24-hour rhythmicity), recent findings showed that cellular NAD^+^ amount also fluctuates throughout the lifespan. Intracellular NAD^+^ has been demonstrated to decrease with aging in humans and mice tissues/cells [44–47]. NAD^+^ amount is estimated to be around 8.54 ± 1.55 ng/mg protein in human newborn (0-1 year) skin tissues but is decreased to 2.74 ± 0.41 and 1.06 ± 0.91 ng/mg protein in young adult (30–50 years) and elderly (>71 years) skin tissues, respectively [46]. Over the last couple of years, many papers have demonstrated that the administration of NAD^+^ precursor, NMN or nicotinamide riboside (NR), to old mice with low cellular NAD^+^ could restore NAD^+^ amount as well as SIRT1 activity, thereby rescuing many metabolic functions [48–52]. Furthermore, NR administration to aging mice, beginning at 2 years of age, could increase mean lifespan with concomitant improvements in maximal running times and distances, along with limb grip strength [49]. A large number of studies in mice show an association between NAD^+^ metabolism and aging. However, studies in humans to address that link are scarce and limited information is available regarding changes in NAD^+^ levels with aging, particularly in human tissues. Further studies are absolutely necessary to understand the relationship between NAD^+^ metabolism and aging process and whether supplementation of NAD^+^ has a benefit for humans against aging/age-related diseases.

While NAMPT is believed to be the enzyme controlling overall cellular NAD^+^ amount, via* Nampt* expression and activation [44, 47], another enzyme was recently reported to be implicated in age-dependent NAD^+^ depletion [53]. CD38, one of the NAD^+^-consuming enzymes, increased in the liver of aged mice and has been shown to deplete NAD^+^ totally by degrading the NAD^+^ precursor, NMN,* in vivo* [53]. Further investigations are required to fully elucidate all the aspects that affect the cellular mechanisms of NAD^+^ reduction with aging.

## 8. Link between Aging and Circadian Clock

Like other physiological events, circadian rhythms have been reported to be attenuated with aging at multiple levels. At the behavior level, locomotor activity rhythms, including free-running period, active mass, and amplitude, are changed with aging, thereby causing sleep fragmentation [54–56]. At the tissue and cellular levels, aging weakens or reduces the amplitude of circadian gene expression patterns [57, 58]. Age-related diseases as well as aging can therefore have adverse effects on circadian clock [59–62]. For instance, mice fed with HFD demonstrate a lengthened free-running period [63], while* db/db* mice, which are an animal model of type 2 diabetes, show attenuated locomotor activity rhythms with diminished* per2 *mRNA and advanced* bmal1 *oscillation [64]. CLOCK has also been reported to be upregulated in human glioma and breast cancer cells, being involved in cancer proliferation [65, 66]. While HFD induces obesity, recent papers clearly indicate that mice fed with HFD along with time-restricted feeding during active period were protected against obesity, hyperinsulinemia, hepatic steatosis, and inflammation with improvement of clock gene expression pattern [67, 68]. This suggests that maintaining circadian metabolic cycles can prevent age-related diseases and probably aging.

On the other hand, many circadian gene knockout mice exhibited accelerated aging with shortened lifespan and/or are prone to develop age-related diseases, including cancer, hypertension, and diabetes.* Bmal1* ^−/−^ mice showed a variety of age-related diseases including sarcopenia, cataracts, decrease of subcutaneous fat, organ shrinkage, dyslipidemia, arterial and venous thrombosis, and hypoinsulinaemia [69–72], with drastically short average lifespan of 37.0 ± 12.1 weeks (wk) [71]. In addition,* clock* ^Δ19/Δ19^ or* clock* ^−/−^ mice displayed age-related diseases such as hyperlipidemia, hyperleptinemia, hyperglycemia, and hypoinsulinaemia with shorter average lifespan of 100.8 ± 13.5 wk, compared to that of wild type mice (115.5 ± 18.9 wk) [70, 73]. In addition to the mouse studies, a large array of epidemiological surveys have provided evidence supporting the link between metabolic disorders, especially obesity, insulin resistance, cardiovascular disease, and cancer, and circadian disruption in humans [62, 74].

Though not yet directly tested, we speculate that circadian NAD^+^ oscillations are diminished or missing in aged animals. The reasons are as follows: (i) as circadian gene expression patterns in aged animal has been demonstrated to be weakened or vanished [57, 58], circadian clock-dependent* Nampt* gene expression patterns may be attenuated; (ii) cellular NAD^+^ amounts in some circadian knockout mice are significantly lower than in wild type mice [30, 31]; (iii) aged animals have much lower cellular NAD^+^, suggesting that even if NAD^+^ amount still shows oscillation, its amplitude could be smaller in aged animals.

Also, since administration of NAD^+^ precursors has shown improvements against age-related diseases and lifespan [48–52], issues to be resolved next are whether cellular NAD^+^ rescued by NAD^+^ precursors in old mice demonstrate an oscillation within 24 hours and improve patterns of circadian gene expressions and chromatin property. These researches will give us physiological significance of circadian NAD^+^ oscillations.

## 9. Concluding Remarks

The accumulating evidence to date reveals the interconnection between circadian clock and aging/age-related diseases [59–62], and, as discussed in this review, NAD^+^ metabolism has a close relationship with both the circadian clock and aging/age-related diseases. However, no direct evidence has so far demonstrated the role of NAD^+^ as the linking hub between circadian clock and aging and age-related diseases. Therefore, this is the obvious area that remains to be explored. More work is needed to reveal how chromatin remodeling through NAD^+^ metabolism takes place. Revealing these connections will open up multiple avenues for the understanding of aging processes and also potential therapeutic interventions.
